# ARID1A loss promotes RNA editing of CDK13 in an ADAR1-dependent manner

**DOI:** 10.1186/s12915-024-01927-9

**Published:** 2024-06-05

**Authors:** Tianyu Zhu, Qian Li, Zhe Zhang, Jiahao Shi, Yongyun Li, Feng Zhang, Lingjie Li, Xin Song, Jianfeng Shen, Renbing Jia

**Affiliations:** 1 Department of Ophthalmology, Ninth People’s Hospital, Shanghai Jiao Tong University School of Medicine, Shanghai, P.R. China; 2 Shanghai Key Laboratory of Orbital Diseases and Ocular Oncology, Shanghai, P.R. China; 3 Department of Histoembryology, Genetics and Developmental Biology, Shanghai Key Laboratory of Reproductive Medicine, Key Laboratory of Cell Differentiation and Apoptosis of Chinese Ministry of Education, Shanghai Jiao Tong University School of Medicine, Shanghai, P.R. China

**Keywords:** ARID1A, RNA editing, ADAR1, CDK13, Tumorigenesis

## Abstract

**Background:**

ARID1A, a subunit of the SWI/SNF chromatin remodeling complex, is thought to play a significant role both in tumor suppression and tumor initiation, which is highly dependent upon context. Previous studies have suggested that ARID1A deficiency may contribute to cancer development. The specific mechanisms of whether ARID1A loss affects tumorigenesis by RNA editing remain unclear.

**Results:**

Our findings indicate that the deficiency of ARID1A leads to an increase in RNA editing levels and alterations in RNA editing categories mediated by adenosine deaminases acting on RNA 1 (ADAR1). ADAR1 edits the CDK13 gene at two previously unidentified sites, namely Q113R and K117R. Given the crucial role of CDK13 as a cyclin-dependent kinase, we further observed that ADAR1 deficiency results in changes in the cell cycle. Importantly, the sensitivity of ARID1A-deficient tumor cells to SR-4835, a CDK12/CDK13 inhibitor, suggests a promising therapeutic approach for individuals with ARID1A-mutant tumors. Knockdown of ADAR1 restored the sensitivity of ARID1A deficient cells to SR-4835 treatment.

**Conclusions:**

ARID1A deficiency promotes RNA editing of CDK13 by regulating ADAR1.

**Supplementary Information:**

The online version contains supplementary material available at 10.1186/s12915-024-01927-9.

## Background

AT-rich binding domain 1A (ARID1A) is a subunit of the SWI/SNF chromatin remodeling complex [1]. ARID1A exerts tumor-suppressive effects by regulating various cellular activities, such as cell cycle evolution, cell proliferation, DNA damage repair, and mitochondrial oxidative stress [2]. *ARID1A* mutations have been identified in many cancers, including ovarian clear cell carcinoma, endometrial cancer, gastric cancer, hepatocellular carcinoma, breast cancer, pancreatic cancer, bladder cancer, and renal cancer [3]. These mutations span the entire gene, with the most common types being nonsense, missense, and frameshift mutations [3]. Most of these mutations result in the loss of ARID1A protein expression. Previous studies on ARID1A have primarily focused on its roles in transcriptional regulation and DNA damage repair [4–6]. Shen et al. discovered that ARID1A deficiency impairs checkpoint activation and DNA double-strand breaks (DSB) repair, rendering cancer cells susceptible to DSB-inducing treatments such as ionizing radiation (IR) and PARP inhibitors [7]. However, whether ARID1A deficiency contributes to tumorigenesis through regulating RNA editing has not been investigated.

RNA editing is a common and crucial mechanism of post-transcriptional RNA modification, playing a role in the initiation and progression of tumors [8–11]. There are two types of RNA editing: the conversion of adenine (A) to hypoxanthine (I) and the transformation of cytosine (C) to uracil (U) [12]. The most prevalent form in mammals is A-to-I editing, involving deamidation at C6 of adenosine, which leads to the conversion of A to I in RNA [12, 13]. A-to-I editing has been proposed to participate in the pathogenesis and progression of cancer [13–15]. A-to-I RNA editing is mediated by adenosine deaminases acting on double-stranded RNA (ADARs), a family of A-to-I RNA editing enzymes. The ADAR family comprises the double-stranded RNA-specific ADAR1, ADAR2, and ADAR3 [16]. ADAR1 exists in two isoforms (p150 and p110) [17] and is primarily responsible for the majority of RNA editing activities [18].

In this study, we revealed that ARID1A deficiency results in an increase in the overall RNA editing level. Loss of ARID1A leads to increased ADAR1-mediated RNA editing of CDK13. Consequently, ARID1A-deficient tumor cells become more susceptible to CDK13 inhibitor treatment. These findings provide novel insights into the role of ARID1A in human cancer and provide a mechanistic foundation for targeting ARID1A-deficient tumors.

## Results

### ARID1A deficiency modulates RNA editing levels and categories

**Fig. 1:**
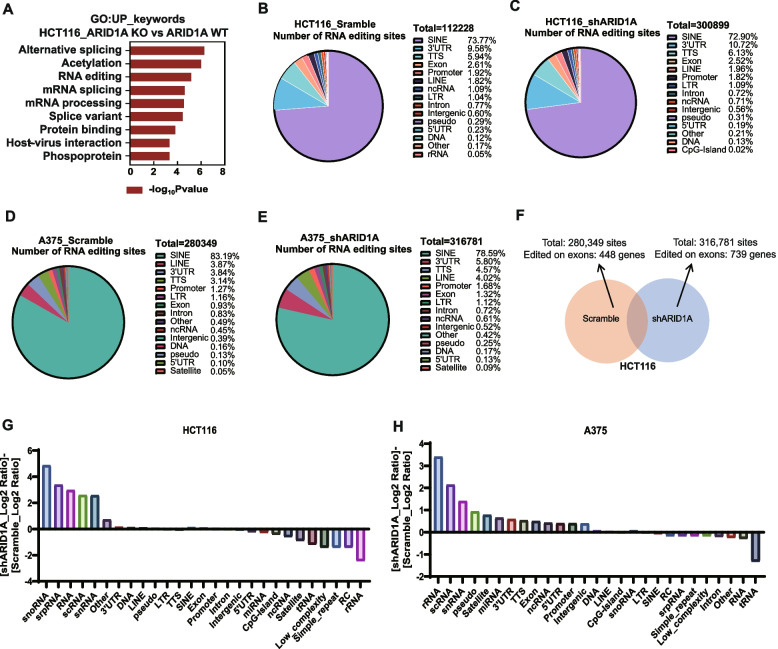
ARID1A deficiency modulates RNA editing levels and types of edited RNAs. **A** GO analysis showing “UP_keywords” category of HCT116_ARID1A_KO compared with ARID1A_WT cell lines. “UP_keywords” = “Uniprot_keywords”. *n* = 2. **B** Pie chart of percentage of A-I RNA editing sites contribution in HCT116_Scramble group. **C** Pie chart of percentage of A-I RNA editing sites contribution in HCT116_shARID1A group. **D** Pie chart of percentage of A-I RNA editing sites contribution in A375_Scramble group. **E** Pie chart of percentage of A-I RNA editing sites contribution in A375_shARID1A group. **F** The amount of A-I RNA editing sites and genes edited on exons between control and ARID1A knockdown A375 cell lines. **G** A-I RNA editing categories show the difference of shARID1A_log2 Ratio and Scramble_log2 Ratio between ARID1A knockdown and control groups in HCT116 cell line. **H** A-I RNA editing categories show the difference of shARID1A_log2 Ratio and Scramble_log2 Ratio between ARID1A knockdown and control groups in A375 cell line

To explore changes in gene enrichment resulting from ARID1A deletion, we performed Gene Oncology analysis on HCT116 ARID1A_WT and ARID1A_KO. Within the “Uniprot_keywords” category, we identified a significant upregulation of “RNA editing” in ARID1A_KO cell lines (Fig. 1A). Volcano plots were employed to depict the differentially expressed genes in the control and ARID1A knockdown group in HCT116 and A375 cell lines (Additional file 1: Fig. S1A-D).

A-I RNA editing manifests across diverse RNA types. In ARID1A_Scramble and shARID1A groups of HCT116, RNA editing predominantly occurred at short interspersed nuclear element (SINE), 3’UTR, and transcription termination sites (TTS) (Fig. 1B-C; Additional file 2: Table S1). In A375 cells, RNA editing was chiefly observed at short interspersed nuclear element (SINE), long interspersed nuclear element (LINE), and 3’UTR (Fig. 1D-E; Additional file 2: Table S2). ARID1A knockdown induced alterations in the distribution and percentage of RNA editing sites.

To further scrutinize the correlation between ARID1A deficiency and RNA editing, we examined the quantity of RNA editing sites in A375 cell lines. In cells with ARID1A deficiency, the number of editing sites increased by over 30,000 and the number of edited exons rose by 291 (Fig. 1F). Log2 ratio (obs / exp) differences between the ARID1A knockdown group and the control group indicated that, at highly edited sites such as SINE and LINE, no significant difference was observed. However, for low-edited sites like rRNA and scRNA, substantial differences existed between the two groups. Furthermore, variations in differential sites were noted between the HCT116 and A375 cell lines (Fig. 1G-H; Additional file 2: Tables S1 and S2).

Collectively, these findings suggest that ARID1A deficiency modulates both RNA editing levels and categories of RNA editing.

### ARID1A deficiency changes RNA editing levels through ADAR1

**Fig. 2:**
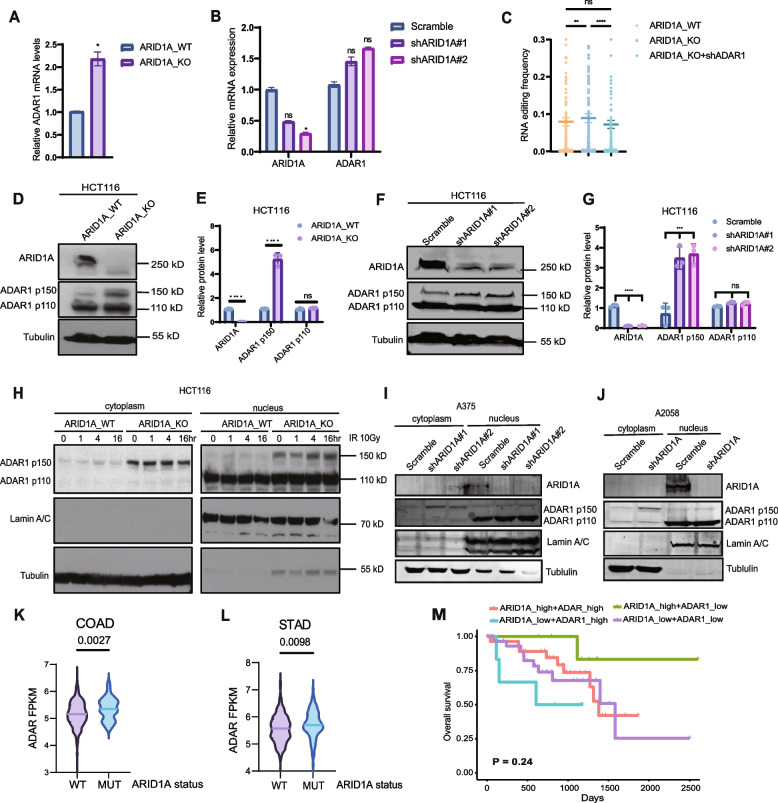
ARID1A deficiency upregulates overall RNA editing level mediated by ADAR1. **A** RT-qPCR results of ADAR1 mRNA expression level in HCT116_ARID1A_WT and HCT116_ARID1A_KO cell lines. *n* = 3; mean ± SD; *, *P* < 0.05. **B** RT-qPCR results of ARID1A and ADAR1 mRNA expression level in HCT116_Scramble, HCT116_shARID1A#1, and HCT116_shARID1A#2 cell lines. *n* = 3; mean ± SD; *, *P* < 0.05; ns, not significant. **C** RNA editing frequency of HCT116_ARID1A_WT, ARID1A_KO, and ARID1A_KO + shADAR1 cell lines. *n* = 2; mean ± SD; **, *P* < 0.01; ****, *P* < 0.0001. ns, not significant. **D** Western blot shows ARID1A and ADAR1 expression in HCT116_ARID1A_WT and HCT116_ARID1A_KO cell lines. **E** Data quantification of protein level of ARID1A, ADAR1 p150 and ADAR1 p110 in HCT116_ARID1A_WT and HCT116_ARID1A_KO cell lines. *n* = 3; mean ± SD; ****, *P* < 0.0001; ns, not significant. **F** Western blot shows ARID1A and ADAR1 expression in HCT116_Scramble, HCT116_shARID1A#1, and HCT116_shARID1A#2 cell lines. **G** Data quantification of protein level of ARID1A, ADAR1 p150 and ADAR1 p110 in HCT116_Scramble, HCT116_shARID1A#1, and HCT116_shARID1A#2 cell lines. *n* = 3; mean ± SD; ***, *P* < 0.001; ****, *P* < 0.0001; ns, not significant. **H** Western blot shows ADAR1 p150 and p110 levels in cytosolic and nuclear fractions in HCT116_ARID1A_WT and HCT116_ARID1A_KO. Cells were treated with IR 10 Gy and fixed at time point of 0 h, 1 h, 4 h, 16 h. **I** Western blot shows ADAR1 p150 and p110 levels in cytosolic and nuclear fractions in A375. **J** Western blot shows ADAR1 p150 and p110 levels in cytosolic and nuclear fractions in A 2058. **K** ADAR1 mRNA expression between ARID1A_WT and ARID1A_MUT in COAD patient specimens (TCGA database). The bar represents the mean ± SD. **L** ADAR1 mRNA expression between ARID1A_WT and ARID1A_MUT in STAD patient specimens (TCGA database). The bar represents the mean ± SD. **M** Kaplan–Meier curves showing TCGA UVM patient overall survival (OS) stratified by tumor ARID1A and ADAR1 mRNA expression. Red: ARID1A_high expression and ADAR_high expression; Green: ARID1A_high expression and ADAR_low expression; Blue: ARID1A_low expression and ADAR_high expression; Purple: ARID1A_low expression and ADAR_low expression

Our findings indicated that ARID1A knockdown increases the number of RNA editing sites and induces changes in RNA editing categories. ADAR1, a pivotal RNA editor catalyzing A-I deamination, was examined for mRNA expression in HCT116 ARID1A knockdown and knockout cell lines. The mRNA levels of ADAR1 were also evaluated post ARID1A knockdown (Fig. 2A-B). In HCT116 ARID1A_KO cells, the overall RNA editing level increased, and ADAR1 knockdown in ARID1A_KO cells reversed this overall editing degree (Fig. 2C), suggesting that the alteration in RNA editing levels upon ARID1A loss is mediated by ADAR1. Next, we observed a significant increase in ADAR1 protein levels, particularly p150 rather than p110, following ARID1A depletion (Fig. 2D-G). Conversely, ADAR2 expression did not show an increase after ARID1A knockdown in HCT116, A375, and A2058 cell lines (Additional file 3: Fig. S2A).

We further investigated whether ARID1A knockdown affected the subcellular distribution of ADAR1 in the nucleus and cytoplasm. Subcellular fractionation analysis revealed an increased cytoplasmic distribution of ADAR1 p150 after ARID1A knockout, while ADAR1 p110 remained concentrated in the nucleus (Fig. 2H). The distribution was unaffected by IR treatment and was consistent in A375 and A2058 (Fig. 2I-J).

Next, we examined the ADAR1 mRNA expression levels in TCGA database between ARID1A_WT and ARID1A_MUT groups. ADAR1 mRNA expression levels in colon adenocarcinoma (COAD) and stomach adenocarcinoma (STAD) patient samples were significantly higher in ARID1A_MUT samples (Fig. 2K-L). Kaplan–Meier curves revealed that UVM patients with low ARID1A expression and high ADAR1 expression exhibited the worst overall survival compared to those with high ARID1A expression, both ARID1A and ADAR1 high expression, and both low expression (Fig. 2M).

In summary, these data illustrate that ARID1A deficiency impacts altered RNA editing levels by upregulating ADAR1 and influences the cellular distribution of ADAR1 p150 and p110.

### ARID1A deficiency slightly changes the editing level of the Q/R site of GlutR-B

**Fig. 3:**
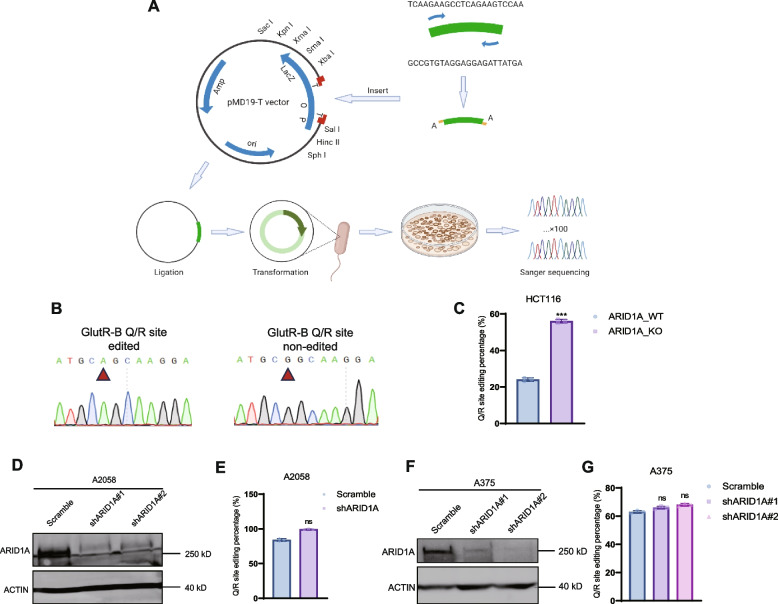
ARID1A deficiency slightly changes the editing level of the Q/R site of GlutR-B. **A** Schematic overview of the TA cloning process. After PCR amplification of a segment of the gene containing the Q/R site of GlutR-B, the fragment was inserted into the T-vector. After ligation and transformation, 100 single clones were selected and plated for Sanger sequencing. Sequence alignment was performed using SnapGene software. **B** Comparison of GlutR-B Q/R site sequences in edited state and non-edited state. The red arrow indicates the Q/R site. **C** The Q/R site editing rate in HCT116 ARID1A_WT and ARID1A_KO cell lines. *n* = 3 of independent experiments; ***, *P* < 0.001. **D** Western blot shows ARID1A knockdown efficiency in A2058 shARID1A cells. **E** The Q/R site editing rate in HCT116 ARID1A_WT and ARID1A_KO cell lines. *n* = 3 of independent experiments; ns, not significant. **F** Western blot shows ARID1A knockdown efficiency in A375 shARID1A cells. **G** The Q/R site editing rate in HCT116 ARID1A_WT and ARID1A_KO cell lines. *n* = 3 of independent experiments; ns, not significant

Our data indicated that ARID1A deficiency leads to an increase in RNA editing levels. Consequently, we sought to investigate whether ARID1A deficiency could influence the RNA editing levels of specific genes. The Q/R site of the GlutR-B gene is among the most well-known RNA edited gene loci. Therefore, we examined whether the deletion of ARID1A resulted in an altered editing rate at this specific location. We designed primers for the GlutR-B Q/R site and amplified the specific fragment (Fig. 3A). The PCR products were subsequently cloned into a TA vector, and Sanger sequencing was performed on 100 colonies. The results of the edited and unedited GlutR-B Q/R sites were analyzed (Fig. 3B). ARID1A deficiency led to higher rates of Q/R site editing at the GlutR-B site in HCT116, A2058, and A375 cell lines (Fig. 3C–G). However, the RNA editing level of the GlutR-B Q/R site only exhibited a slight increase after ARID1A deficiency. This suggests that while ARID1A deficiency impacts the overall RNA editing level, it does not play a predominant role in the editing level of the GlutR-B Q/R site.

### The CDK13 gene can be edited by ADAR1 and ARID1A deficiency increased sensitivity to SR-4835

**Fig. 4:**
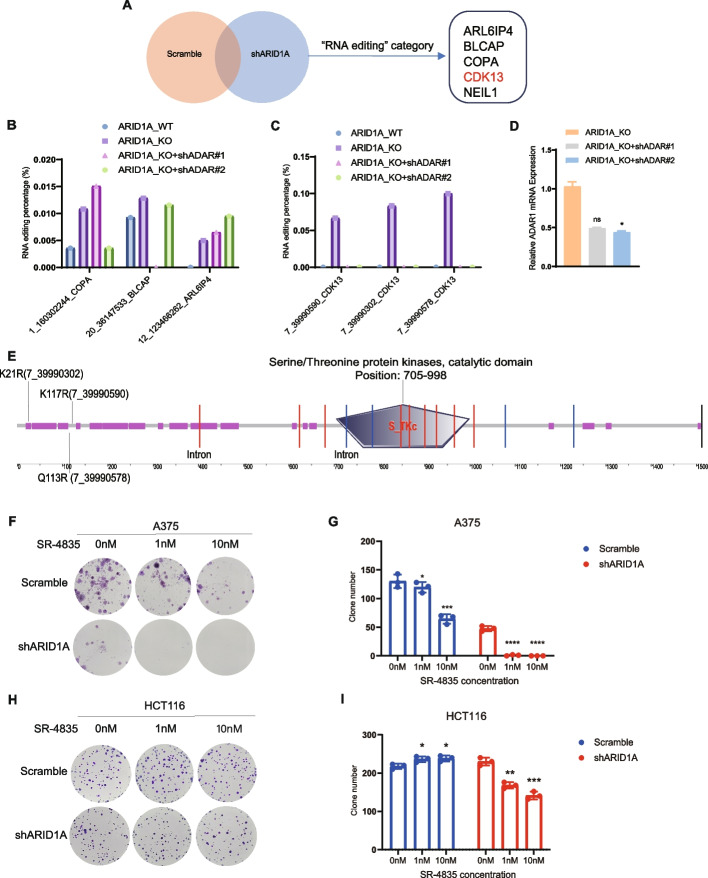
The CDK13 gene is edited by ADAR1 and ARID1A deficiency increased sensitivity to SR4835. **A** Comparison of HCT116 shARID1A to ARID1A_Scramble cell lines. The differential genes in “RNA editing” category consists of ARL6IP4, BLCAP, COPA, CDK13, and NEIL1. **B** The RNA editing frequency at different sites of COPA, BLCAP, ARL6IP4 genes in HCT116 ARID1A_WT, ARID1A_KO, ARID1A_KO + shADAR#1, and ARID1A_KO + shADAR#2 cell lines. **C** The RNA editing frequency at three different sites of CDK13 genes in HCT116 ARID1A_WT, ARID1A_KO, ARID1A_KO + shADAR#1, and ARID1A_KO + shADAR#2 cell lines. **D** RT-qPCR results show ADAR mRNA expression in HCT116 ARID1A_KO, ARID1A_KO + shADAR#1, and ARID1A_KO + shADAR#2 cell lines. *n* = 3; mean ± SD; *, *P* < 0.05; ns, not significant. **E** Diagram of the CDK13 kinase, showing the serine/threonine protein kinase active region. Intron regions and three RNA editing sites (K21R, K117R, and Q113R) are marked. K21R:7_39990302; K117R:7_39990590; Q113R: 7_39990578. Exported by "SMART" software, the red line represents intron Phase 2 and the blue line represents intron Phase 1. **F** Representative images of A375 Scramble and shARID1A cells treated with SR-4835 at a concentration of 1 nM and 10 nM. **G** Quantitative results represent the mean ± SD of three independent experiments. *, *P* < 0.05; ***, *P* < 0.001; ****, *P* < 0.0001. **H** Representative images of HCT116 Scramble and shARID1A cells treated with SR-4835 at a concentration of 1 nM and 10 nM. **I** Quantitative results represent the mean ± SD of three independent experiments. *, *P* < 0.05; **, *P* < 0.01; ***, *P* < 0.001

We proceeded to investigate whether other genes were subject to increase editing by ADAR1. Our bioinformatic analysis revealed an overall increase in RNA editing rates and modifications in the types of edited RNAs in cells with ARID1A knockdown. Moreover, the “RNA editing” category exhibited differences between the ARID1A knockdown and control groups. Genes within the RNA editing category, including ARL6IP4, BLCAP, COPA, CDK13, and NEIL1, were found to be altered by ARID1A knockdown, suggesting changes in the RNA editing levels of these genes after ARID1A deficiency (Fig. 4A). Consequently, we compared the RNA editing rates of genes in the ARID1A_WT, ARID1A_KO, ARID1A_KO + shADAR#1, and ARID1A_KO + shADAR#2 groups. The editing rate in COPA, BLCAP, and ARL6IP4 did not increase after ARID1A KO and decreased after ADAR1 knockdown (Fig. 4B).

Results indicated a simultaneous increase in the RNA editing rate of the CDK13 gene at three loci, 7_39990302_CDK13_ + _Nonsyn_Lys- > Arg (K21R), 7_39990578_CDK13_ + _ Nonsyn_Gln- > Arg (Q113R), and 7_39990590_CDK13_ + _Nonsyn_Lys- > Arg (K117R) after ARID1A knockout, signifying a substantial rise in RNA editing of CDK13 gene following ARID1A deletion. Upon the combination of ADAR1 knockdown, the RNA editing level of CDK13 mediated by ARID1A deletion decreased, indicating that the change in RNA editing level of CD13 is achieved through ADAR1 (Fig. 4C). The knockdown efficiency of ADAR1 was verified by RT-qPCR (Fig. 4D). The three editing sites were marked on the CDK13 protein, all of which were distant from catalytic domain (Fig. 4E).

Considering that CDK13 plays a crucial role in cell cycle and participates in RNA editing, we selected CDK13 for further investigation. ARID1A deletion enhanced RNA editing of CDK13, potentially compromising its biological activity. We then explored whether the CDK13 inhibitor could be employed to assess the role of CDK13 and whether the therapeutic effect was influenced by ARID1A. Various CDK13 inhibitors, including SR-4835, highly selective for CDK12 and CDK13 with an ATP-competitive binding mechanism, were considered. It interacts with the kinase’s hinge region by hydrogen bonding [19]. SR-4835 exhibits anticancer activity in ovarian cancer, triple-negative breast cancer, and leukemia [19–21]. In the absence of pharmacological treatment, ARID1A knockdown markedly decreased tumor clone development; under SR-4835 treatment, ARID1A-deficient cells demonstrated improved sensitivity to CDK13 inhibitors (Fig. 4F-I; Additional file 4: Fig. S3A-C).

### The Q113R and K117R of CDK13 are edited by ADAR1 and ADAR1 knockdown rescued sensitivity to SR-4835

**Fig. 5:**
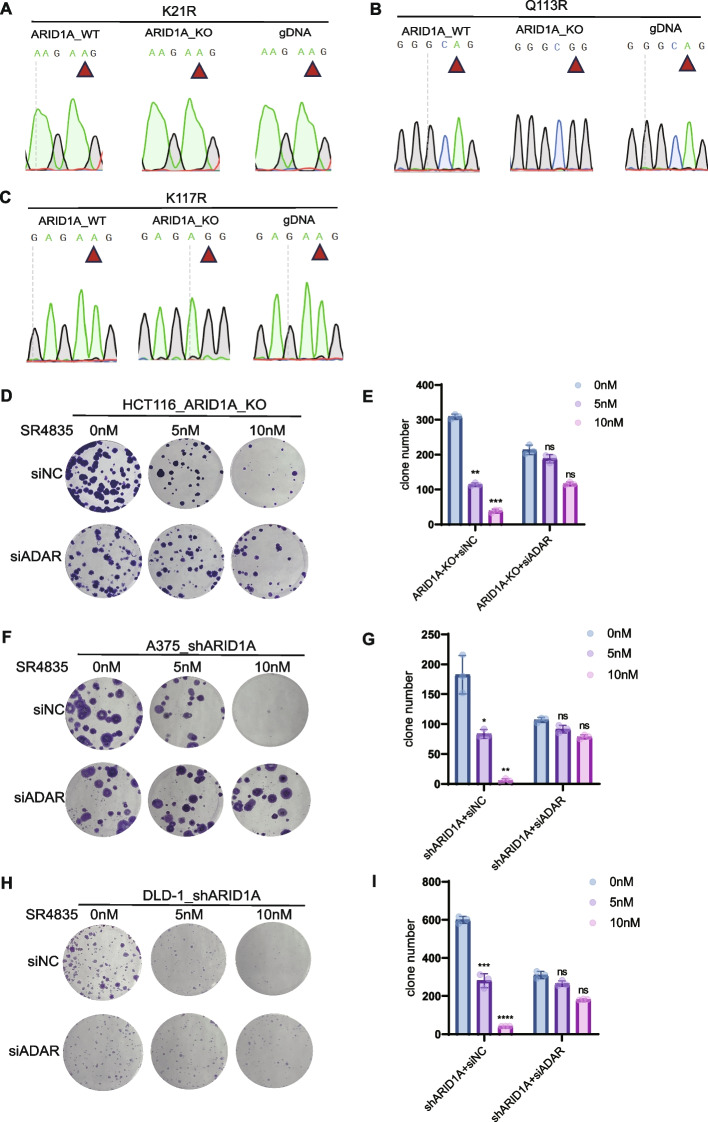
ADAR1 edits CDK13 at position Q113R and K117R, ADAR1 knockdown rescues the sensitivity to SR4835. **A** Sanger sequencing chromatograms using a reverse primer illustrate editing of CDK13 K21R position in ARID1A_WT, ARID1A_KO and genomic DNA (gDNA) in HCT116 cell lines. **B** Sanger sequencing chromatograms using a reverse primer illustrate editing of CDK13 Q113R position in ARID1A_WT, ARID1A_KO and genomic DNA (gDNA) in HCT116 cell lines. **C** Sanger sequencing chromatograms using a reverse primer illustrate editing of CDK13 K117R position in ARID1A_WT, ARID1A_KO and genomic DNA (gDNA) in HCT116 cell lines. **D** Representative images of HCT116_ARID1A_KO cell lines transfected with siNC and siADAR and treatment with SR-4835 at a concentration of 5 nM and 10 nM. **E** Quantitative results represent the mean ± SD of three independent experiments. **, *P* < 0.01; ***, *P* < 0.001. **F** Representative images of A375_ARID1A_KO cell lines transfected with siNC and siADAR and treatment with SR-4835 at a concentration of 5 nM and 10 nM. **G** Quantitative results represent the mean ± SD of three independent experiments. *, *P* < 0.05; **, *P* < 0.01. **H** Representative images of DLD-1_ARID1A_KO cell lines transfected with siNC and siADAR and treatment with SR-4835 at a concentration of 5 nM and 10 nM. **I** Quantitative results represent the mean ± SD of three independent experiments. ***, *P* < 0.001.****, *P* < 0.0001

In the subsequent experiments, we conducted RT-PCR and Sanger sequencing to validate the RNA editing status at the K21R, Q113R, and K117R sites, as identified in the bioinformatics analysis. No corresponding edits were detected at the K21R site (Fig. 5A). Notably, at the Q113R and K117R positions, A-to-G editing events were observed following ARID1A knockout (Fig. 5B-C).

Furthermore, when ARID1A KO was combined with siNC, cells displayed heightened sensitivity to SR4835. Conversely, when combined with ADAR1 knockdown, cells exhibited reduced sensitivity to SR4835 treatment across HCT116, A375, and DLD-1 cell lines (Fig. 5D-I). This not only supports the observed correlation between ARID1A’s interaction with ADAR but also underscores its impact on the effectiveness of the CDK13 inhibitor.

### ADAR1 deficiency results in altered cell cycle in response to ionizing radiation (IR)

Next, we investigated the influence of ADAR1 on the cell cycle following ARID1A deletion. First, we assessed the cell cycle distribution of ARID1A_WT and ARID1A_KO cells at different time points post-IR. At 4 and 8 h after irradiation, both ARID1A_WT and ARID1A_KO cells exhibited a significant increase in the G2-M population (Additional file 4: Fig. S3D; Additional file 5: Fig. S4A–B). At 16 h post irradiation, a substantial proportion of ARID1A_WT cells remained arrested at the G2-M checkpoint, while the G2M checkpoint arrest in ARID1A-depleted cells was notably reduced. Simultaneously, there was a significant increase in the proportion of cells in the G1 phase.

Upon introducing ADAR1 knockdown in ARID1A_WT cells, we observed a decrease in the proportion of cells in the G1 phase and a more rapid entry into G2-M phases at 4 and 8 h post IR. However, this altered distribution disappeared when ARID1A knockout was simultaneously applied, suggesting that ADAR1 deletion induces changes in the cell cycle in ARID1A-deficient cells. Additionally, ADAR1 knockdown resulted in an increased G2-M arrest in the context of ARID1A-KO.

To further assess the impact on mitotic cells, we utilized phospho-Histone H3 staining. In ADAR1-knockdown ARID1A_KO cells exposed to IR, we observed an increase in cells re-entering to M phase, especially under Taxol treatment. However, this increase was not observed following combined ARID1A deficiency (Additional file 5: Fig. S4C). Collectively, these results suggest that ADAR1 plays a role in cell cycle distribution, indicating potential implications for RNA editing of CDK13. Notably, knockdown of CDK13 in HCT116_ARID1A_WT cells led to a decrease in the G1 phase and an increase in the G2-M phase, which was enhanced upon concomitant ARID1A KO (Additional file 4: Fig. S3E; Additional file 5: Fig. S4D-E).

## Discussion

According to our research, ARID1A plays a role in regulating RNA editing and tumorigenesis, dependent on ADAR1. Our findings highlight that ARID1A deficiency results in heightened RNA editing level and alterations in the profile of edited RNA types. Furthermore, we identified CDK13 as a critical gene subjected to ADAR1-mediated editing. Intriguingly, our data suggests that cells deficient in ARID1A exhibit increased sensitivity to CDK13 inhibitor.

In the current work, we delved into the role of ADAR1, an enzyme central to RNA editing. Upon reviewing RNA sequencing data from other publications, we observed that a twofold increase in the mRNA expression of ADAR1 upon ARID1A loss, corroborating our own findings [22]. While the augmented expression of ADAR1 expression in the ARID1A-deficient context, the precise mechanism linking ARID1A and ADAR1 remains elusive. There is a suspicion that ARID1A deficiency may impact DNA damage, and the relationship between A-to-I editing mediated by ADAR1 and DNA damage has been explored in previous studies [22, 23]. The influence of ARID1A deletion extends beyond altering ADAR1 expression; it also affects the subcellular localization of the two ADAR1 isoforms, p150 and p110. ADAR1 p110, primarily located in the nucleus, engages in editing double-stranded RNA (dsRNA) in the absence of infection. Conversely, ADAR1 p150, is an immune response protein that localizes to the cytoplasm and is expressed later in the infection process [24]. Following ARID1A deletion, we observed an increase in both forms of ADAR1 (p150 and p110) in the nucleus and an increase of p150 in the cytosol. We suspect that ARID1A deficiency might alter the expression of IFN-γ–responsive genes [25], resulting in a shift in the intracellular distribution of ADAR1 p150 and p110. But the conjecture requires further investigation [24].

ARID1A loss caused an increase in the total RNA editing rate and changes in RNA editing categories. Most of the RNA editing sites located in SINE and 3'UTR regions, consistent with prior studies [26, 27]. RNA editing exhibits a dual role, either promoting or inhibiting cancer development, with numerous RNA editing genes and loci identified across different cancer types [28, 29]. GlutR-B, a well-known RNA edited gene, experiences reduced editing at the Q/R site in brain tumors [30–32]. Our data, however, did not show a significant increase in GlutR-B editing after ARID1A deletion. We propose two potential explanations for this observation. First, GlutR-B is primarily edited and regulated by ADAR2, while our data indicates a prominent role for ADAR1 in this process. Second, the limited impact of ARID1A knockdown on the Q/R site in melanoma cell lines could be attributed to the already high editing levels in the Scramble group of these cell lines. This heightened editing in the Scramble group might be influenced by the elevated expression of GRIA2, with additional molecular regulations possibly involved, necessitating further investigation. These findings prompted the identification of novel editing sites for ADAR1 following ARID1A deficiency in the transcriptome.

We further observed an increased rate of RNA editing in the CDK13 gene following ARID1A deletion. *CDK13*, a member of cyclin-dependent kinases (CDKs), plays major roles in regulating the cell cycle and transcription. Previous study has demonstrated high levels of RNA editing in the *CDK13* gene in hepatocellular carcinoma [33]. Elevated CDK13 editing events have been detected in several tumor types, including glioblastoma [34] and kidney renal clear cell carcinoma [14]. CDK13 was recently identified with editing sites at Q103R in thyroid tumors [35, 36]. Notably, our findings unveiled distinct editing sites (K21R, Q113R, and K117R) compared to previously reported sites. Prior research has shown that downregulating CDK13 suppresses colony formation in a breast cancer cell line [19]. Consistent with these findings, our experimental system demonstrated a similar phenomenon with CDK13 inhibitors, indicating that CDK13 could serve as a novel therapeutic target in HCT116 and A375 cell lines. Additionally, ARID1A-deficient cells exhibited sensitivity to CDK13 inhibitor SR-4835, and ADAR1 deletion rescues the sensitivity. It is noteworthy that ARID1A knockdown promotes proliferation in HCT116 and DLD-1 while inhibiting proliferation in the A375 cell line. Recent literature suggests that ARID1A may have context-dependent oncogenic effects. For example, in a mouse model of colon cancer with Apc inactivation, Arid1a is considered essential for tumorigenesis [22]. Despite ARID1A knockdown suppressing proliferation in A375, there remains a stronger sensitivity to CDK13 inhibitors. Therefore, ARID1A-mutant tumors may be promising candidates for CDK13 inhibitor treatment, and ADAR1 involves in this process.

A prior study demonstrated that loss of ARID1A leads to impaired checkpoint activation [7], a finding that aligns with our data. Since we found ADAR1 can edit *CDK13*, which indicates its role on regulating cell cycle. Specifically, ADAR1 impacts the cell cycle distribution, which is consistent with some existing studies [37, 38]. After irradiation, ADAR1 knockdown causes a decrease in G1 phase cells and increased number of cells in G2-M phases. However, ARID1A deficiency enhances ADAR1 expression and modifies this phenomenon further. Therefore, we hypothesize that ARID1A’s regulation of cell cycle may be mediated through ADAR1, but further verification is needed.

## Conclusions

Our findings demonstrate that ARID1A deficiency leads to increased RNA editing activity on CDK13. To the best of our knowledge, our findings provide the first evidence establishing a connection between ARID1A and alterations in RNA editing.

## Methods

### Cell lines and cell culture

The HCT116, DLD-1, A375, and A2058 cell lines were obtained from by American Type Culture Collection (ATCC) and Cell Bank and Stem Cell Bank, Chinese Academy of Sciences. HCT116 ARID1A-knockout cell lines were obtained from Horizon Discovery, Inc [7]. All cell lines were cultured in DMEM (Invitrogen) supplemented with 10% fetal bovine serum (Gibco) and 1% penicillin/streptomycin (Gibco) at 37 °C with 5% CO_2_.

### Antibodies and reagents

Anti-ARID1A (1:1000 dilution in western blot) and anti-ADAR1 (1:1000) antibodies were purchased from Cell Signaling Technology (Danvers, USA). Anti-ACTIN (1:2000) was purchased from Proteintech (Chicago, USA). Anti-LaminA/C (1:1000) was purchased from Cell Signaling Technology. Anti-Tubulin (1:1000) was purchased from Abcam. The CDK13 inhibitor SR-4835 was purchased from Selleckchem (Houston, USA).

### shRNA interference

The shRNA oligos targeting ARID1A and ADAR were inserted into a PLKO.1-based retroviral vector (PLKO.1-Puro) constitutive expression of puromycin resistance. The shRNA sequences were as follows: ARID1A (#1), CCGTTGATGAACTCATTGGTT; ARID1A (#2), CCTCTCTTATACACAGCAGAT; ADAR1 (#1), CCGGCGGATACTACACCCATCCATTCTCGAGAATGGATGGGTGTAGTATCC GTTTTTG; and ADAR1 (#2), CCGGGCCCACTGTTATCTTCACTTTCTCGAGAAAGTGAAGAT AACAGTGGGCTTTTTG. After transduction and puromycin selection, the specificity and efficacy of the ARID1A shRNA and ADAR1 shRNA programs were assessed by western blotting.

### siRNA interference

For siRNA transfection, non-targeting control siRNA (siNC) and siRNA specific for ADAR1 (siADAR1) and CDK13 (siCDK13) were purchased from Zorun Biotechnology Co., Ltd (Shanghai, China).

### Western blot

Cells were harvested and lysed in urea lysis buffer supplemented with protease and phosphatase inhibitors (Sangon Biotech, Shanghai, China) for 30 min at 4 °C. Lysates were centrifuged at 15,000 × g for 15 min at 4 °C. The supernatant fraction was collected and incubated in 5 × SDS-PAGE loading buffer (BioRad, California, USA) at 95 °C for 10 min for protein denaturation. Equivalent aliquots of protein samples (20–40 µg) were electrophoresed on SDS-PAGE gels and then transferred to PVDF membranes (Millipore, Darmstadt, Germany). Membranes were blocked in 5% non-fat milk (Sangon Biotech) for 1 h at room temperature. The membranes were then incubated overnight at 4 °C with primary antibodies diluted in TBST at overnight. Membranes were washed with PBST and incubated with Secondary Antibody (1:10,000; Licor, Lincoln, USA) diluted in TBST. Finally, membranes were washed with TBST, and the bound antibodies were detected by enhanced chemiluminescence (Odyssey CLx, Lincoln, USA).

### Subcellular fractionation

Nuclear and cytosolic fractions were obtained utilizing the Nuclear and Cytoplasmic Extraction Kit (CWBIO, Yunnan, China). Cells were harvested and resuspended in 1 ml of Nc-buffer A and 55 µl of Nc-buffer B, then incubated on ice for 20 min. Samples were centrifuged at 12,000 × g for 15 min, and the supernatants which contain the cytoplasmic component and pellets containing nuclei were used for protein extraction.

### TA cloning

To compare the RNA editing rate of the Q/R site of GlutR-B, we amplified a fragment including the Q/R site from cDNA. SuperScript II reverse transcriptase (Vazyme, Nanjing, China) was used to convert total RNA into cDNA with oligo d(T). The PCR products were purified using a gel extraction purification kit (Vazyme) and then cloned into the pMD-19-T vector (Takara, Kyoto, Japan) following the manufacturers’ instructions. The vectors were transformed into DH5α competent cells (Tsingke Biotechnology Co., Ltd., Beijing, China), and a total of 100 clones were randomly selected for Sanger sequencing on an ABI 3730 DNA sequence. The rate of Q/R site editing was calculated by dividing the total number of clones by the number of clones that showed editing.

### Sanger sequencing to verify CDK13 editing sites

The verification of the three RNA editing sites, K21R, Q113R, and K117R was conducted through reverse transcription polymerase chain reaction (RT-PCR) using the following PCR primers. For K21R: Forward (F): ATGCCGAGCAGCTCGGACA and Reverse (R): CAGCCAGGAAGAGCAGAGG. For Q113R and K117R: F: GCGGAGAAGAAGTTGGAGGA and R: ATCCTCGTATTCCACCAGCG. The PCR products were subsequently subjected to validation through Sanger sequencing to confirm the accuracy and specificity of the RNA editing events at these identified sites.

### RNA isolation and quantitative RT-PCR

Total RNA was isolated from cells using the EZBioscience Kit, and cDNA was synthesized using SuperScript III Reverse Transcriptase (Vazyme). Quantitative PCR was performed in a LightCycler PCR instrument (Roche, Basel, Switzerland) using SYBR Green Master Mix (Vazyme). All reactions were performed in triplicate. The PCR primer sequences for qRT-PCR are listed in Additional file 2: Table S3. Gene expression was normalized to levels of human ACTB. Fold change in expression levels were calculated using the 2^−ΔΔCT^ method. Relative expression levels and standard deviations were calculated using the comparative method.

### RNA sequencing

For HCT116 and A375 cell lines, ARID1A Scramble and two shRNA hairpins were performed for RNA sequencing (*n* = 2 / group). HCT116_ARID1A_WT and HCT116_ARID1A_KO were performed for RNA sequencing (*n* = 2 / group). The data has been uploaded the data to the Figshare database (10.6084/m9.figshare.25016681). Total RNA was isolated using Trizol reagent (Invitrogen), and its concentration, quality and integrity were determined using a NanoDrop spectrophotometer (Thermo Scientific, Wilmington, USA). Sequencing libraries were generated using the TruSeq RNA Sample Preparation Kit (Illumina, California, USA). mRNA was purified from total RNA using magnetic beads bound to oligo poly-T and fragmented at high temperature using divalent cations in Illumina proprietary fragmentation buffer. In a 15-cycle PCR reaction, a cocktail of Illumina PCR Primer was used to selectively enrich DNA fragments with inducer molecules bound at both ends. Products were purified (AMPure XP system, Indiana, USA) and quantified on a Bioanalyzer 2100 system (Agilent) using the highly sensitive Agilent DNA assay. Sequencing libraries were then sequenced on an Illumina Novaseq 6000 by Shanghai Personal Biotechnology Cp. Ltd. Gene Ontology analysis was performed using the program DAVID. Volcano plots are used to illustrate the upregulated and downregulated differentially expressed genes in the control and ARID1A knockdown group.

### RNA editing analysis

We used SPRINT (https://sprint.tianlab.cn) to identify RNA editing sites using RNA-seq data [39]. Filtering focused on A-to-I editing events, detecting adenosine (A) to inosine (I) conversions. Following identification, the RNA editing levels were quantified by calculating the ratio of edited reads to total reads at each site, generating a comprehensive profile across the transcriptome. Differential RNA editing analysis utilized Fisher's exact test, identifying significantly differentially edited sites based on adjusted *p*-values.

### Colony formation assay

Cells were seeded in 12-well plates at a density of 500 cells per well. The next day, cells were treated with corresponding inhibitors or vehicle as indicated. Cells were cultured and the medium was changed every 2–3 days. Two weeks later, cells were fixed with 4% buffered paraformaldehyde and stained with 0.01% crystal violet.

### Statistical analysis

All statistical analyses were performed using GraphPad Prism 9.0 software. Three independent experiments were performed to check the reproducibility of each experiment. Bars represent the means ± SD. Two-tailed Student’s t-tests and one-way ANOVA were used to evaluate the data.

### Supplementary Information

**Additional file 1: Figure S1.** Volcano plots of ARID1A knockdown and control groups in HCT116 and A375 cell lines. (A) The volcano plot of HCT116_shARID1A#1 compared to Scramble illustrates significant changes in gene expression, with upregulated (red), downregulated (blue) and not-significant (grey) genes. *n*=2. (B) The volcano plot of HCT116_shARID1A#2 compared to Scramble illustrates significant changes in gene expression, with upregulated (red), downregulated (blue) and not-significant (grey) genes. *n*=2. (C) The volcano plot of A375_shARID1A#1 compared to Scramble illustrates significant changes in gene expression, with upregulated (red), downregulated (blue) and not-significant (grey) genes. *n*=2. (D) The volcano plot of A375_shARID1A#2 compared to Scramble illustrates significant changes in gene expression, with upregulated (red), downregulated (blue) and not-significant (grey) genes. *n*=2.

**Additional file 2: Table S1-S3.** Table S1. A-I RNA editing sites of shARID1A and Scramble in HCT116. Table S2. A-I RNA editing sites of shARID1A and Scramble in A375. Table S3. Primers used in this study for RT-qPCR.

**Additional file 3: Figure S2.** Western blot of ARID1A, ADAR1 and ADAR2 expression of ARID1A knockdown and control cell lines. (A) Western blot indicates ARID1A, ADAR1 and ADAR2 expression in HCT116 ARID1A _WT, HCT116_ARID1A_KO, A375_Scramble, A375_shARID1A#1, A375_shARID1A#2, A2058_Scramble, A2058_shARID1A#1 and A2058_shARID1A#2 cell lines.

**Additional file 4: Figure S3.** ARID1A deficiency increased sensitivity to SR4835 in DLD-1. (A) Representative images of DLD-1 Scramble and shARID1A cells treated with SR-4835 at a concentration of 1 nM and 10 nM. (B) Quantitative results represent the mean ± SD of three independent experiments. *, *P*<0.05; ***, *P* <0.001. (C) RT-qPCR results of ARID1A mRNA expression level in DLD-1_Scramble, DLD-1_shARID1A cell lines. *n*=3; mean ± SD; *, *P*<0.05. (D) RT-qPCR results of ADAR1 mRNA expression level in ARID1A_WT and ARID1A_KO with or without siADAR1 cell lines. *n*=3; mean ± SD; *, *P* < 0.05; **, *P* < 0.01. (E) RT-qPCR results of CDK13 mRNA expression level in ARID1A_WT and ARID1A_KO with or without siCDK13 cell lines. *n*=3; mean ± SD; *, *P* < 0.05.

**Additional file 5: Figure S4.** ADAR1 and CDK13 deficiency changes the cell cycle in response to ionizing radiation (IR). (A) HCT116 ARID1A_WT cells transfected with Scramble and ADAR1 siRNA were exposed to IR (7Gy). Cells were fixed at different time points and tested cell cycle distribution. *n*=2. (B) HCT116 ARID1A_KO cells transfected with Scramble and ADAR1 siRNA were exposed to IR (7Gy). Cells were fixed at different time points and tested cell cycle distribution. *n*=2. (C) Phospho-Histone H3 (pH3) was determined at the indicated time points after irradiation. *n*=3. (D) HCT116 ARID1A_WT cells transfected with siNC and siCDK13 were exposed to IR (7Gy). Cells were fixed at different time points and tested cell cycle distribution. *n*=3; mean±SD. *n*=2. (E) HCT116 ARID1A_KO cells transfected with siNC and siCDK13 were exposed to IR (7Gy). Cells were fixed at different time points and tested cell cycle distribution. *n*=3; mean±SD. *n*=2.

**Additional file 6: Figure S5.** Images of the full immunoblots.

**Additional file 7.** The individual data values for Fig. 2A, 2B, 2E, 2G, 3C, 3E, 3G, 4B, 4C, 4D, 4G, 4I, 5E, 5G, 5I, S3A, S3B, S3C, S3E, S4B, S4C, S4D, S4E.

## Data Availability

All data generated or analysed during this study are included in this published article, its supplementary information files and publicly available repositories. The individual data values of the replicates are listed in Additional file 7. Sequencing datasets are available via Figshare (10.6084/m9.figshare.25016681).

## Abbreviations

DSB: DNA double-strand breaks
IR: Ionizing radiation
ADAR: Adenosine deaminases acting on double-stranded RNA
SINE: Short interspersed nuclear element
TTS: Transcription termination sites
COAD: Colon adenocarcinoma
STAD: Stomach adenocarcinoma
dsRNA: Double-stranded RNA
CDKs: Cyclin-dependent kinases
