# Supplementary figures and images for: ARID1A loss promotes RNA editing of CDK13 in an ADAR1-dependent manner
Source: BMC Biol. 2024 Jun 5;22:132. doi: 10.1186/s12915-024-01927-9 (PMC11151582; doi:10.1186/s12915-024-01927-9)

Figure S1

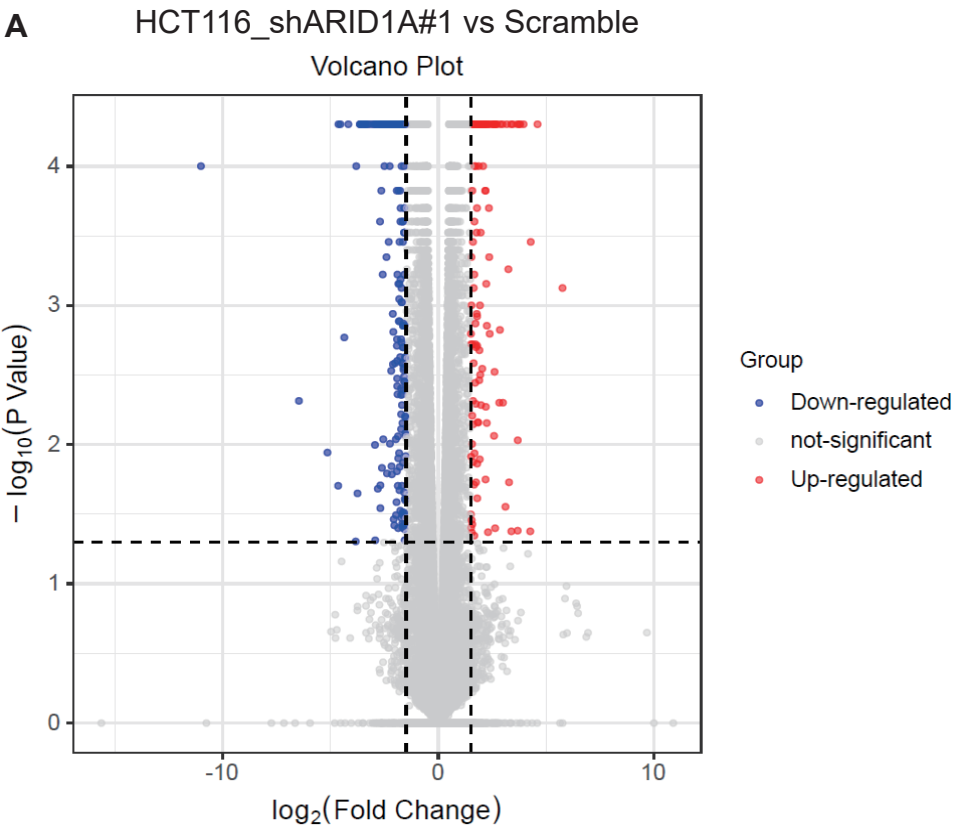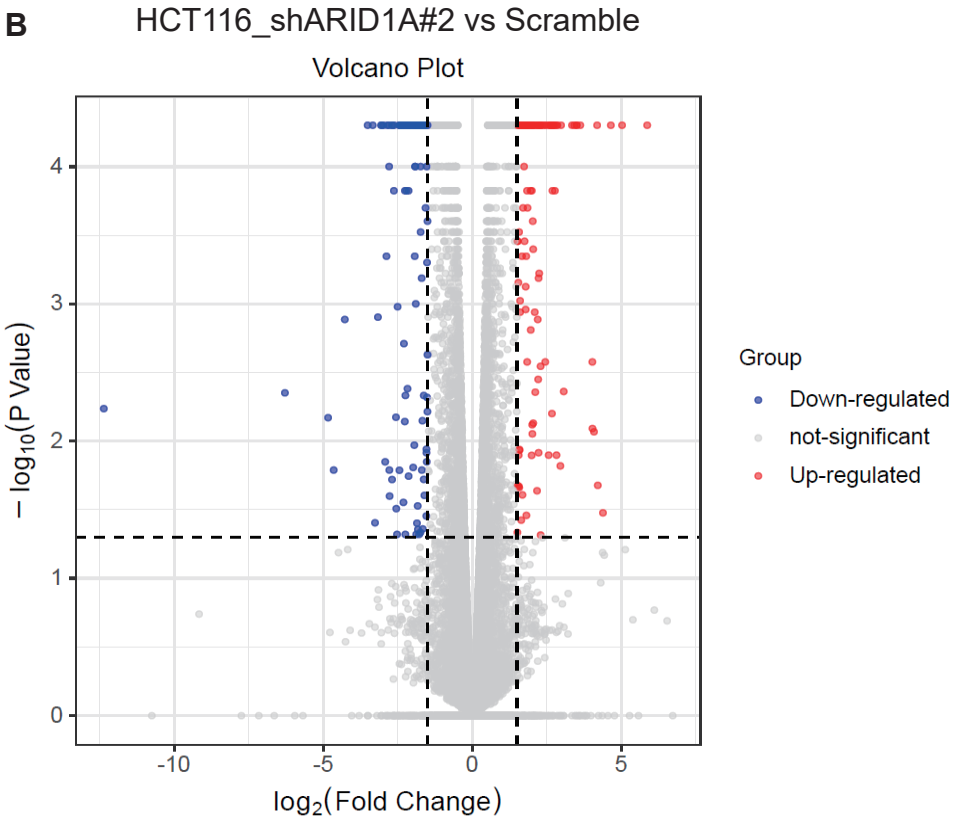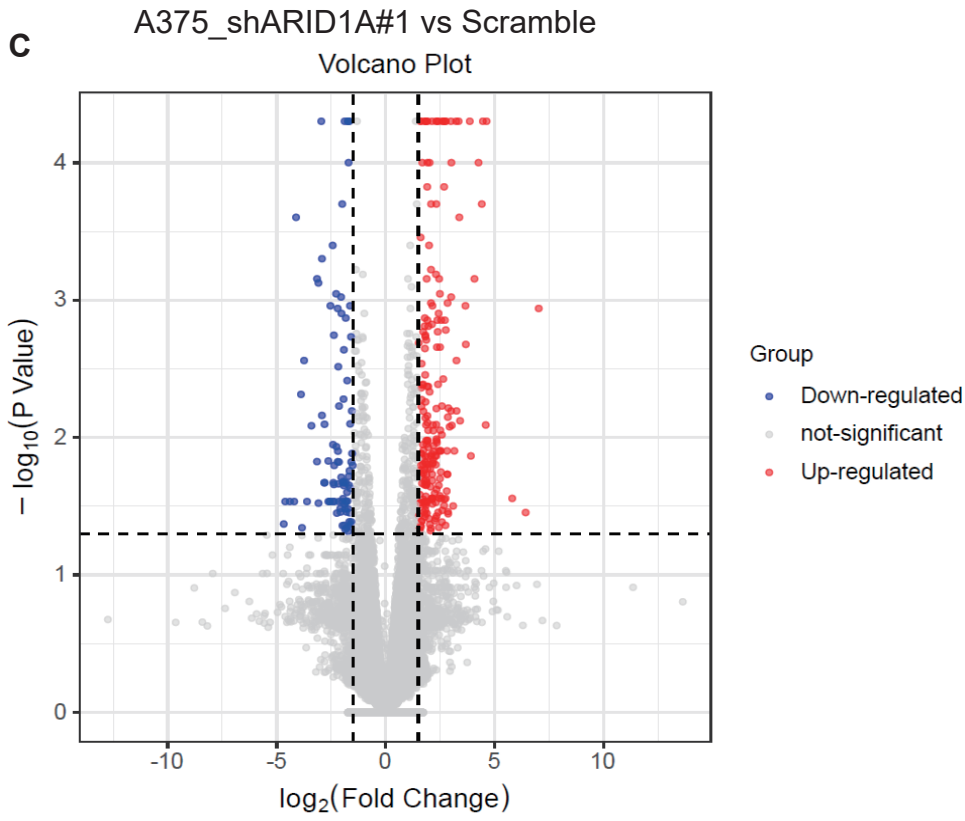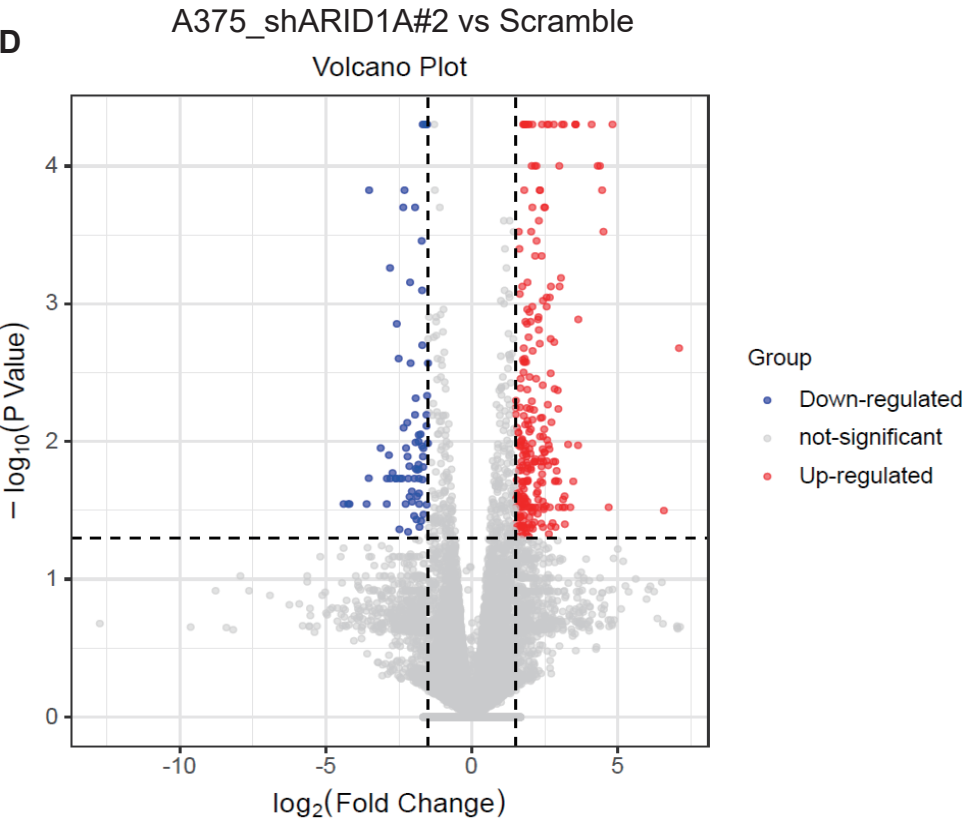

Supplement: Supplementary file 1 — Additional file 1: Figure S1. Volcano plots of ARID1A knockdown and control groups in HCT116 and A375 cell lines. (A) The volcano plot of HCT116_shARID1A#1 compared to Scramble illustrates significant changes in gene expression, with upregulated (red), downregulated (blue) and not-significant (grey) genes. n=2. (B) The volcano plot of HCT116_shARID1A#2 compared to Scramble illustrates significant changes in gene expression, with upregulated (red), downregulated (blue) and not-significant (grey) genes. n=2. (C) The volcano plot of A375_shARID1A#1 compared to Scramble illustrates significant changes in gene expression, with upregulated (red), downregulated (blue) and not-significant (grey) genes. n=2. (D) The volcano plot of A375_shARID1A#2 compared to Scramble illustrates significant changes in gene expression, with upregulated (red), downregulated (blue) and not-significant (grey) genes. n=2. [file 12915_2024_1927_MOESM1_ESM.pdf]

Figure S2

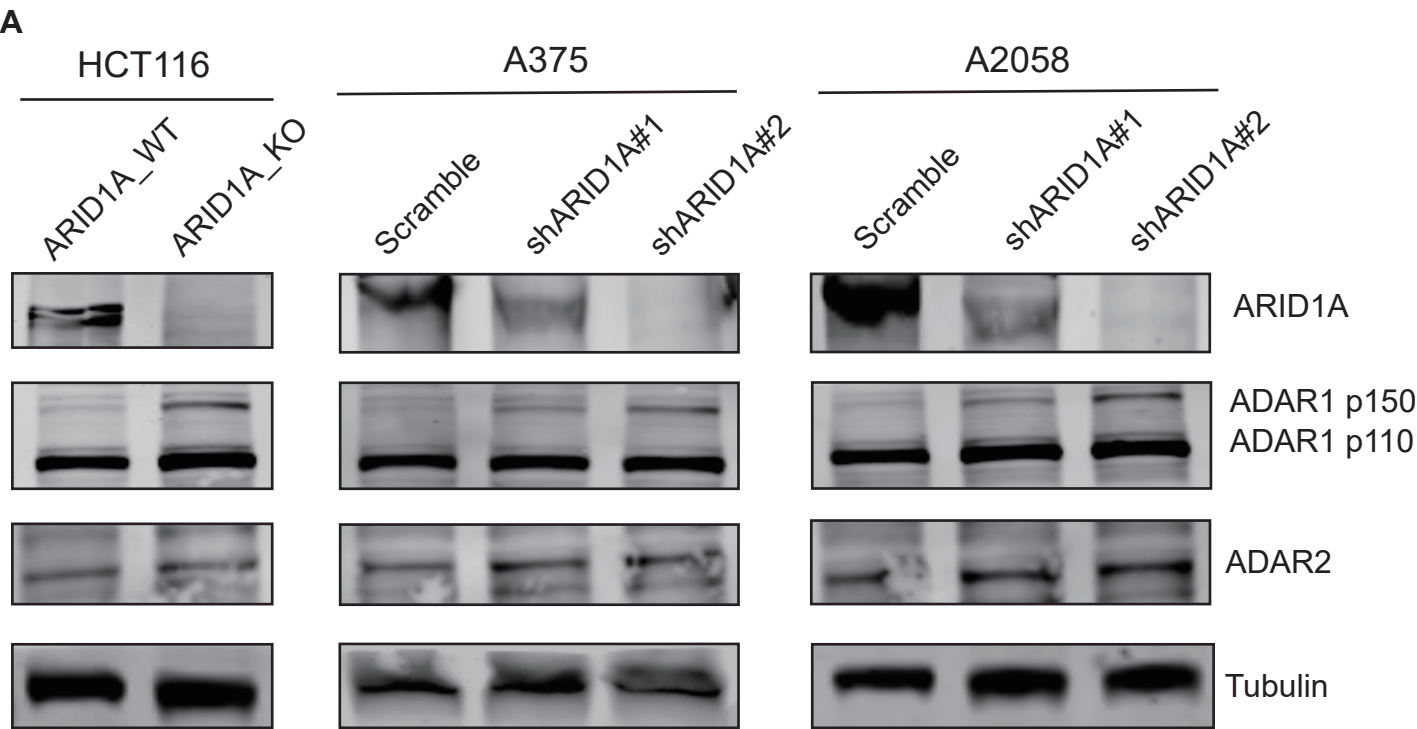

Supplement: Supplementary file 3 — Additional file 3: Figure S2. Western blot of ARID1A, ADAR1 and ADAR2 expression of ARID1A knockdown and control cell lines. (A) Western blot indicates ARID1A, ADAR1 and ADAR2 expression in HCT116 ARID1A _WT, HCT116_ARID1A_KO, A375_Scramble, A375_shARID1A#1, A375_shARID1A#2, A2058_Scramble, A2058_shARID1A#1 and A2058_shARID1A#2 cell lines. [file 12915_2024_1927_MOESM3_ESM.pdf]

Figure S3

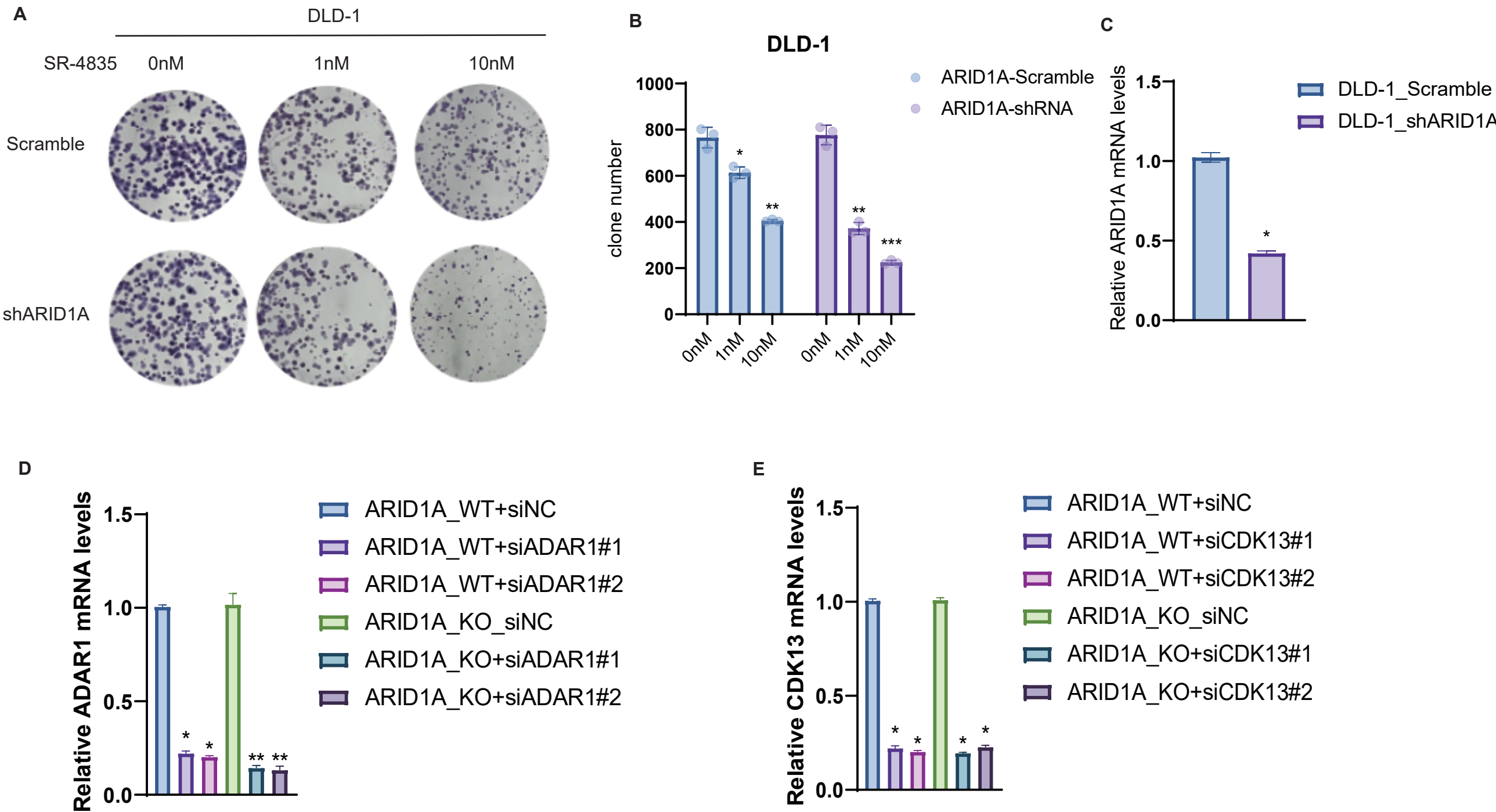

Supplement: Supplementary file 4 — Additional file 4: Figure S3. ARID1A deficiency increased sensitivity to SR4835 in DLD-1. (A) Representative images of DLD-1 Scramble and shARID1A cells treated with SR-4835 at a concentration of 1 nM and 10 nM. (B) Quantitative results represent the mean ± SD of three independent experiments. *, P<0.05; ***, P <0.001. (C) RT-qPCR results of ARID1A mRNA expression level in DLD-1_Scramble, DLD-1_shARID1A cell lines. n=3; mean ± SD; *, P<0.05. (D) RT-qPCR results of ADAR1 mRNA expression level in ARID1A_WT and ARID1A_KO with or without siADAR1 cell lines. n=3; mean ± SD; *, P < 0.05; **, P < 0.01. (E) RT-qPCR results of CDK13 mRNA expression level in ARID1A_WT and ARID1A_KO with or without siCDK13 cell lines. n=3; mean ± SD; *, P < 0.05. [file 12915_2024_1927_MOESM4_ESM.pdf]

Figure S4

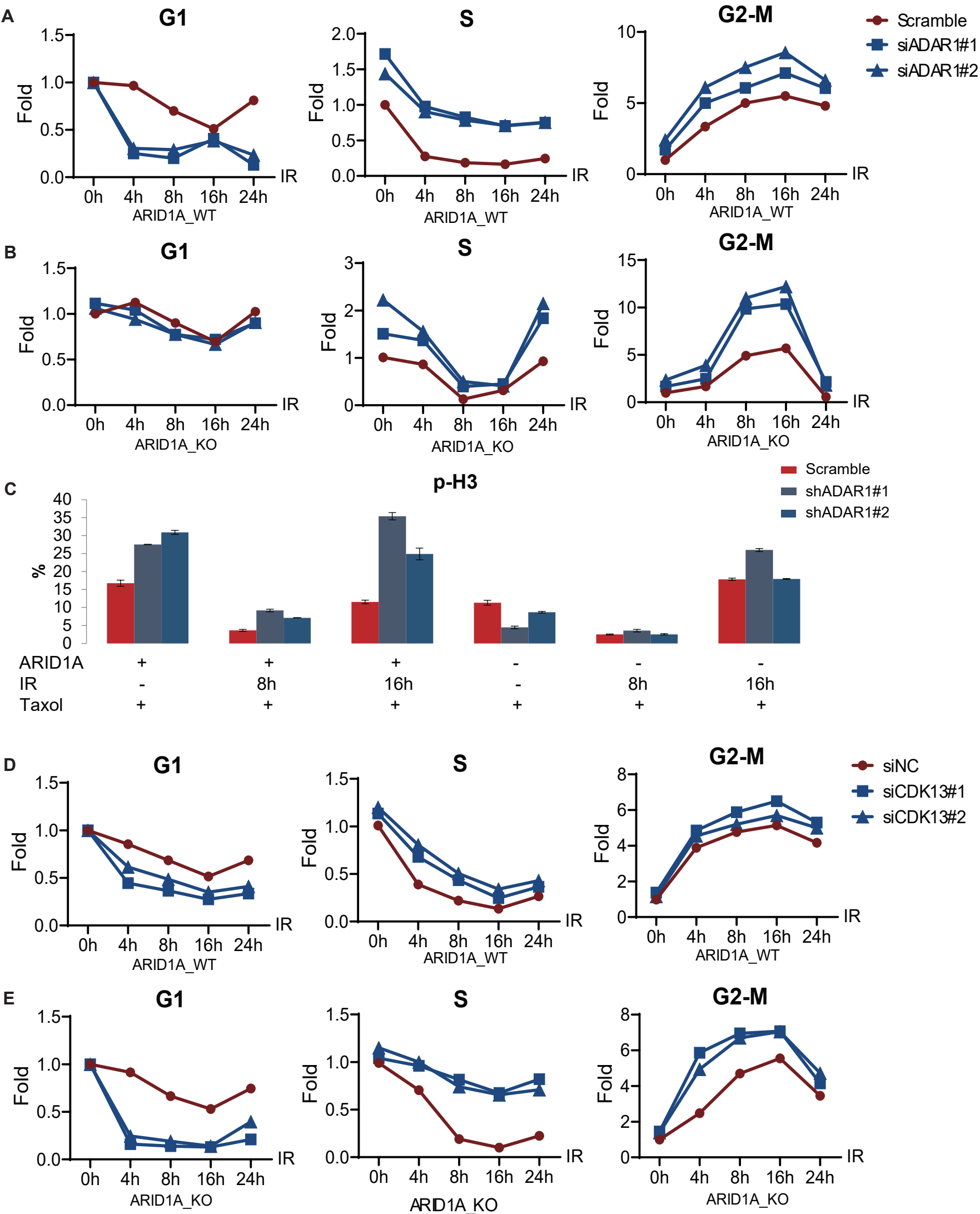

Supplement: Supplementary file 5 — Additional file 5: Figure S4. ADAR1 and CDK13 deficiency changes the cell cycle in response to ionizing radiation (IR). (A) HCT116 ARID1A_WT cells transfected with Scramble and ADAR1 siRNA were exposed to IR (7Gy). Cells were fixed at different time points and tested cell cycle distribution. n=2. (B) HCT116 ARID1A_KO cells transfected with Scramble and ADAR1 siRNA were exposed to IR (7Gy). Cells were fixed at different time points and tested cell cycle distribution. n=2. (C) Phospho-Histone H3 (pH3) was determined at the indicated time points after irradiation. n=3. (D) HCT116 ARID1A_WT cells transfected with siNC and siCDK13 were exposed to IR (7Gy). Cells were fixed at different time points and tested cell cycle distribution. n=3; mean±SD. n=2. (E) HCT116 ARID1A_KO cells transfected with siNC and siCDK13 were exposed to IR (7Gy). Cells were fixed at different time points and tested cell cycle distribution. n=3; mean±SD. n=2. [file 12915_2024_1927_MOESM5_ESM.pdf]

**Additional file 6:**

**
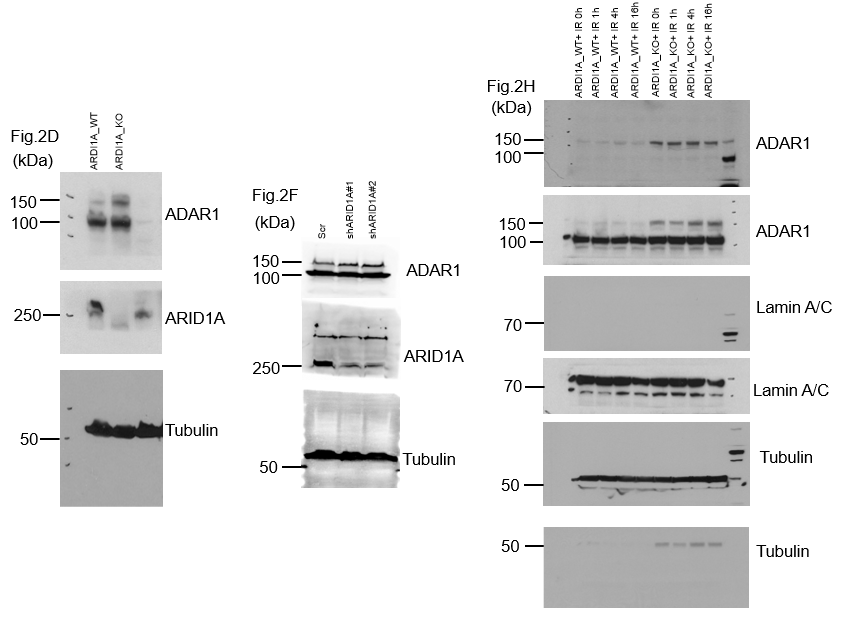
**

**
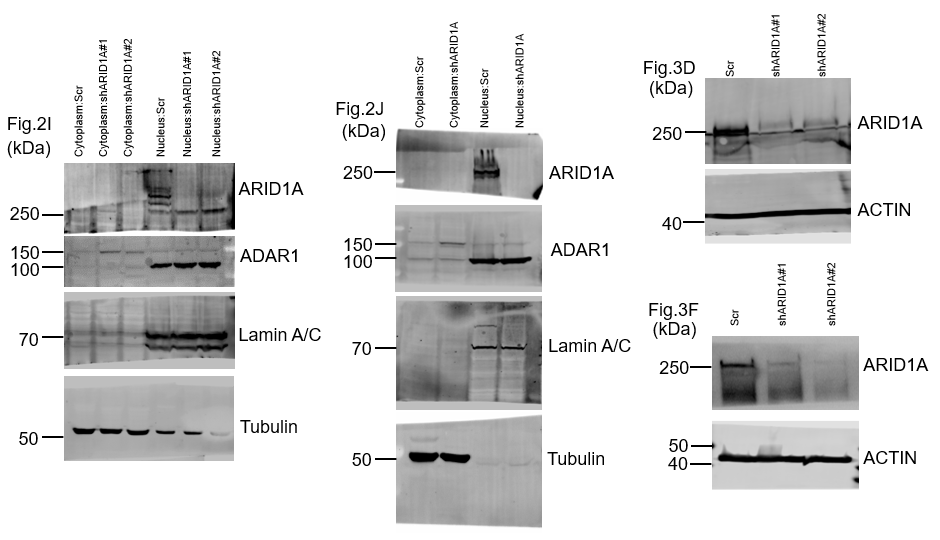
**

**
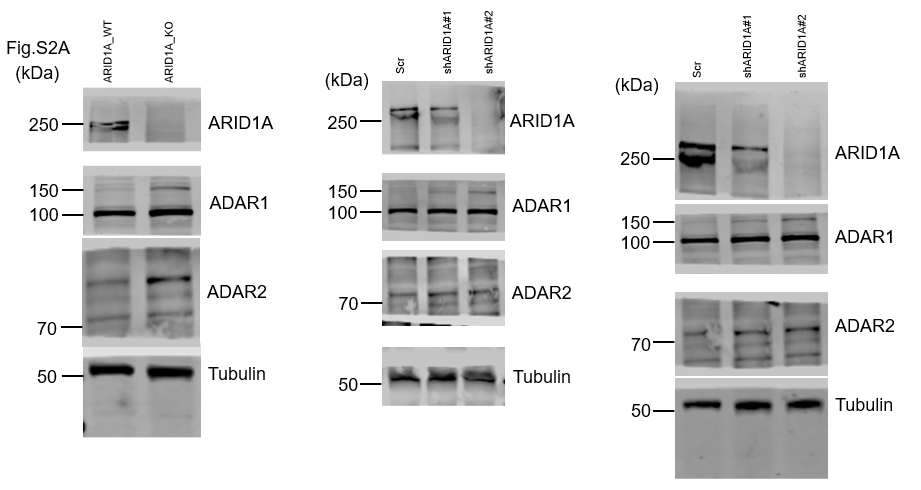
**

**Figure S5. Images of the full immunoblots.**

Supplement: Supplementary file 6 — Additional file 6: Figure S5. Images of the full immunoblots. [file 12915_2024_1927_MOESM6_ESM.docx]
