# Supplementary material for: ARID1A loss promotes RNA editing of CDK13 in an ADAR1-dependent manner
Source: BMC Biol. 2024 Jun 5;22:132. doi: 10.1186/s12915-024-01927-9 (PMC11151582; doi:10.1186/s12915-024-01927-9)
Supplement: Supplementary file 2 — Additional file 2: Table S1-S3. Table S1. A-I RNA editing sites of shARID1A and Scramble in HCT116. Table S2. A-I RNA editing sites of shARID1A and Scramble in A375. Table S3. Primers used in this study for RT-qPCR. [file 12915_2024_1927_MOESM2_ESM.docx]

**Table S1**

|  |  | **HCT116_Scr** | | **HCT116_shARID1A** | | **shARID1A-Scr** |
| --- | --- | --- | --- | --- | --- | --- |
| **Annotation** | **Total size (bp)** | **Number of sites** | **Log2 Ratio (obs/exp)** | **Number of sites** | **Log2 Ratio (obs/exp)** | **Log2 - Log2** |
| 3UTR | 26762632 | 10751 | 3.47 | 32258 | 3.632 | 0.162 |
| Other | 3935577 | 104 | -0.456 | 461 | 0.269 | 0.725 |
| RNA | 114374 | 0 | -2.364 | 17 | 0.613 | 2.977 |
| miRNA | 96670 | 10 | 1.513 | 22 | 1.227 | -0.286 |
| ncRNA | 6991458 | 1220 | 2.267 | 2137 | 1.653 | -0.614 |
| TTS | 32286633 | 6669 | 2.51 | 18441 | 2.555 | 0.045 |
| LINE | 622739191 | 2046 | -3.464 | 5911 | -3.356 | 0.108 |
| srpRNA | 252957 | 1 | -3.197 | 28 | 0.187 | 3.384 |
| SINE | 378584817 | 82787 | 2.593 | 219341 | 2.575 | -0.018 |
| RC | 442102 | 5 | -1.681 | 5 | -3.104 | -1.423 |
| tRNA | 91526 | 0 | -2.111 | 0 | -3.307 | -1.196 |
| pseudo | 2089637 | 329 | 2.119 | 932 | 2.198 | 0.079 |
| DNA | 95640101 | 132 | -4.715 | 390 | -4.575 | 0.14 |
| Exon | 37006581 | 2930 | 1.127 | 7584 | 1.076 | -0.051 |
| Intron | 664015350 | 866 | -4.797 | 2173 | -4.893 | -0.096 |
| Intergenic | 863542451 | 678 | -5.529 | 1696 | -5.629 | -0.1 |
| Promoter | 35809872 | 2156 | 0.732 | 5488 | 0.657 | -0.075 |
| 5UTR | 2586029 | 253 | 1.432 | 570 | 1.181 | -0.251 |
| snoRNA | 357 | 0 | -0.019 | 1 | 4.849 | 4.868 |
| scRNA | 115467 | 0 | -2.375 | 13 | 0.212 | 2.587 |
| CpG-Island | 8588734 | 30 | -3.376 | 60 | -3.799 | -0.423 |
| Low_complexity | 15393271 | 0 | -9.134 | 0 | -10.555 | -1.421 |
| LTR | 259145993 | 1168 | -3.008 | 3273 | -2.944 | 0.064 |
| Simple_repeat | 24806381 | 0 | -9.826 | 0 | -11.248 | -1.422 |
| snRNA | 313162 | 1 | -3.505 | 16 | -0.928 | 2.577 |
| Unknown | 1244230 | 0 | -5.528 | 0 | -6.931 | -1.403 |
| Satellite | 12339025 | 39 | -3.52 | 56 | -4.421 | -0.901 |
| rRNA | 164138 | 53 | 3.155 | 26 | 0.704 | -2.451 |

**Table S2**

|  |  | **A375_Scr** | | **A375_shARID1A** | | **shARID1A-PLKO.1** |
| --- | --- | --- | --- | --- | --- | --- |
| **Annotation** | **Total size (bp)** | **Number of sites** | **Log2 Ratio (obs/exp)** | **Number of sites** | **Log2 Ratio (obs/exp)** | **Log2 - Log2** |
| rRNA | 164138 | 1 | -3.894 | 12 | -0.485 | 3.409 |
| scRNA | 115467 | 1 | -3.386 | 5 | -1.241 | 2.145 |
| snRNA | 313162 | 2 | -3.826 | 6 | -2.417 | 1.409 |
| pseudo | 2089637 | 360 | 0.928 | 782 | 1.871 | 0.943 |
| Satellite | 12339025 | 150 | -2.897 | 293 | -2.108 | 0.789 |
| miRNA | 96670 | 14 | 0.677 | 25 | 1.337 | 0.66 |
| 3UTR | 26762632 | 10769 | 2.152 | 18358 | 2.745 | 0.593 |
| TTS | 32286633 | 8802 | 1.59 | 14477 | 2.131 | 0.541 |
| Exon | 37006581 | 2600 | -0.366 | 4177 | 0.141 | 0.507 |
| ncRNA | 6991458 | 1260 | 0.993 | 1920 | 1.424 | 0.431 |
| 5UTR | 2586029 | 281 | 0.263 | 420 | 0.666 | 0.403 |
| Promoter | 35809872 | 3560 | 0.134 | 5313 | 0.536 | 0.402 |
| Intergenic | 863542451 | 1104 | -6.147 | 1646 | -5.747 | 0.4 |
| DNA | 95640101 | 455 | -4.251 | 546 | -4.164 | 0.087 |
| LINE | 622739191 | 10840 | -2.379 | 12722 | -2.325 | 0.054 |
| CpG-Island | 8588734 | 63 | -3.626 | 73 | -3.59 | 0.036 |
| snoRNA | 357 | 0 | -0.046 | 0 | -0.052 | -0.006 |
| LTR | 259145993 | 3264 | -2.846 | 3563 | -2.896 | -0.05 |
| SINE | 378584817 | 233217 | 2.766 | 248948 | 2.684 | -0.082 |
| RC | 442102 | 0 | -5.359 | 0 | -5.531 | -0.172 |
| Simple_repeat | 24806381 | 0 | -11.146 | 0 | -11.322 | -0.176 |
| srpRNA | 252957 | 10 | -1.196 | 10 | -1.372 | -0.176 |
| Low_complexity | 15393271 | 0 | -10.453 | 0 | -10.63 | -0.177 |
| Unknown | 1244230 | 1 | -6.816 | 0 | -7.004 | -0.188 |
| Intron | 664015350 | 2322 | -4.695 | 2274 | -4.901 | -0.206 |
| Other | 3935577 | 1270 | 1.833 | 1211 | 1.588 | -0.245 |
| RNA | 114374 | 1 | -3.373 | 0 | -3.667 | -0.294 |
| tRNA | 91526 | 2 | -2.051 | 0 | -3.374 | -1.323 |

| **Table S3. Primers used in this study** | | |
| --- | --- | --- |
| **Primer for RT-qPCR** | | |
| **Target Gene** | **Forward Primer 5'-3'** | **Reverse Primer 5'-3'** |
| *ARID1A* | CCTGAAGAACTCGAACGGGAA | TCCGCCATGTTGTTGGTGG |
| *ADAR1* | CTGAGACCAAAAGAAACGCAGA | GCCATTGTAATGAACAGGTGGT |
| *ADAR1 p150* | CGGGCAATGCCTCGC | AATGGATGGGTGTAGTATCCGC |
| *ADAR1 p110* | GACTGAAGGTAGAGAAGGCTACG | TGCACTTCCTCGGGACAC |
| *CDK13* | CCCCTAGTCCCTACAGCAG | GCCTAGATGAATACGGGCTTCTG |
| *ACTB* | CATGTACGTTGCTATCCAGGC | CTCCTTAATGTCACGCACGAT |
